# Distinct Cell Stress Responses Induced by ATP Restriction in Quiescent Human Fibroblasts

**DOI:** 10.3389/fgene.2016.00171

**Published:** 2016-10-04

**Authors:** Nirupama Yalamanchili, Andres Kriete, David Alfego, Kelli M. Danowski, Csaba Kari, Ulrich Rodeck

**Affiliations:** ^1^School of Biomedical Engineering, Science and Health Systems, Drexel University, PhiladelphiaPA, USA; ^2^Department of Dermatology, St. Joseph Mercy Health System, Michigan State University, East LansingMI, USA; ^3^Department of Dermatology and Cutaneous Biology, Thomas Jefferson University, PhiladelphiaPA, USA

**Keywords:** metabolism, aging, inflammation, NF-κB signaling, Akt, p53, ischemia, mTOR pathway

## Abstract

Quiescence is the prevailing state of many cell types under homeostatic conditions. Yet, surprisingly little is known about how quiescent cells respond to energetic and metabolic challenges. To better understand compensatory responses of quiescent cells to metabolic stress, we established, in human primary dermal fibroblasts, an experimental ‘energy restriction’ model. Quiescence was achieved by short-term culture in serum-deprived media and ATP supply restricted using a combination of glucose transport inhibitors and mitochondrial uncouplers. In aggregate, these measures led to markedly reduced intracellular ATP levels while not compromising cell viability over the observation period of 48 h. Analysis of the transcription factor (TF) landscape induced by this treatment revealed alterations in several signal transduction nodes beyond the expected biosynthetic adaptations. These included increased abundance of NF-κB regulated TFs and altered TF subsets regulated by Akt and p53. The observed changes in gene regulation and corresponding alterations in key signaling nodes are likely to contribute to cell survival at intracellular ATP concentrations substantially below those achieved by growth factor deprivation alone. This experimental model provides a benchmark for the investigation of cell survival pathways and related molecular targets that are associated with restricted energy supply associated with biological aging and metabolic diseases.

## Introduction

Metabolic challenges due to limitations in nutrient uptake or mitochondrial dysfunction trigger adaptations that ultimately determine cell fate and survival. A key nodal point of metabolic adaption upon nutrient deprivation is induction of autophagy via inhibition of the serine/threonine kinase mammalian target of rapamycin (mTOR) (Kim et al., 2002; Laplante and Sabatini, 2009). In addition, metabolic challenges common in disease and aging are also accompanied by adaptive changes in TF activity, as exemplified by increased Nuclear Factor kappaB (NF-κB) transcriptional activity. NF-κB activation and increased expression of genes encoding inflammatory proteins has been observed in aged cells and tissues (Adler et al., 2007; Kriete et al., 2008; Kriete and Mayo, 2009; Salminen and Kaarniranta, 2009; Tilstra et al., 2011). It has also been suggested that energy stress caused by dysfunctional mitochondria may contribute to NF-κB induction in mammalian cells reminiscent of the retrograde response in yeast (Biswas et al., 2008; Srinivasan et al., 2010; Jazwinski and Kriete, 2012).

Much of the current understanding of metabolic stress responses is based on work with cycling cultured cells, or, in the case of aging, with cells subjected to replicative senescence (Toussaint et al., 2000; Bernard et al., 2004; Coppe et al., 2008; Chien et al., 2011). Metabolic dysfunction of fibroblasts under conditions resembling physiological tissue conditions has only recently come into focus (Koziel et al., 2011; Tigges et al., 2014). For many tissues including fibroblasts the prevalent cell state in the organism is quiescence (Yao, 2014). However, responses to metabolic perturbations in quiescent cells are often more subtle and difficult to detect when compared to metabolically active and cycling cells *in vitro*. To mimic quiescence and low biosynthetic rates prevalent *in vivo*, we limited in the present study the exogenous growth factor supply to cultured human fibroblasts for 24 h (serum deprivation). In addition, we depleted intracellular ATP levels in these cells by the combined use of mitochondrial respiration uncouplers and a glucose utilization inhibitor for further 24 h. At non-toxic levels, these interventions caused a marked decrease in ATP concentrations. Genome-wide transcriptional profiling revealed a distinct shift in the TF landscape in response to combined nutrient and ATP deprivation. Changes in transcriptional regulation pointed to activated NF-κB and Akt signaling nodes while p53 expression and signaling was reduced. Our results establish an experimental approach to dissect signaling networks in energy-deprived quiescent cells and highlight distinct signaling nodes that may contribute to cell survival in metabolically challenged cells and tissues.

## Materials and Methods

### Cell Line and Culture Procedures

The human fibroblast culture (AG10803, Coriell Institute for Medical Research, Camden, NJ) used in this study was derived from a 2 mm punch biopsy taken from the abdomen of a young donor. AG10803 cells have a normal karyotype. For experiments, low-passage (<6 population doublings) non-senescent cells were used. Cells were grown in medium consisting of EMEM (Mediatech, Herndon, VA) supplemented with 2 mM L-glutamine and 15% FBS without antibiotics at 37°C and 5% CO_2_. 24 h prior to experiments, cells were placed in growth factor-free, serum- free medium (MEM supplemented with 0.2% Bovine Serum Albumin/BSA), see Supplementary Figure S1. In order to reduce intracellular ATP levels, drugs that target the two major sources of ATP in the cell were added to the medium for another 24 h. To reduce glycolysis, we used 2-Deoxy-D-glucose (2DG) (Sigma, D6134) at 5 and 10 mM. 2DG was dissolved in water to prepare a 1M stock solution. In addition, mitochondrial respiration inhibitors were used to uncouple the respiration chain from the phosphorylation reaction. Uncoupling agents tested included 2, 4-Dinitrophenol (DNP) and Carbonyl cyanide 4-(trifluoromethoxy)phenylhydrazone (FCCP), which were used at working concentrations of 5 mM and 10 μM, respectively. DNP (Sigma, D198501) and FCCP (Sigma, C2920) were dissolved in 95% ethanol to prepare 833 and 50 mM stock solutions, respectively. Inhibitors were applied in different concentrations and combinations to achieve a gradual ATP depletion. At the end of the experiments, after 48 h, samples were collected and downstream assays performed as described below.

### Immunoblotting and Quantification

For immunoblot analysis, 30–45 μg of protein from acetone precipitated cytoplasmic extracts were resolved by gel electrophoresis using 4–20% gradient gels followed by transfer to nitrocellulose membranes. Primary antibodies to detect proteins were as follows: phospho-Akt (Ser473), Akt, phospho-GSK3β (S9), IκB-α, Phospho-Bad (Ser136) (D25H8), LC3B, ATG5, and phosphor-p70S6K (Thr389) (Cell Signaling cat # 9271, 9272, 9336, 9242, 4366, 2775, 2630, and 9206, respectively). Other primary antibodies include anti-NF-κB p65 (phospho S276) (from Abcam ab30623), p62 (BML-PW9860 Enzo Life Sciences), p53 (sc-263 Santa Cruz Biotechnology, Inc.), phosphor-eIF2α (Ser52) (sc-101670 Santa Cruz Biotechnology, Inc.), and GSK3β (610201 BD Trans. Lab.). The blots were stripped and reprobed 3–4 times. All blots were probed with α-tubulin (CP06, Calbiochem) for loading controls. Immunoblots were scanned and band intensities were measured using ImageJ (NIH Image, RSB). These measurements were normalized to α-tubulin loading controls. Readouts from experiments were averaged, standard deviations determined and *t*-tests, where applicable, were performed using MS Excel.

### Cell Cycle Analysis

5 – 6 × 10^5^ cells were seeded in a 100 mm petri dish with 16 ml growth medium. After 24 h, the growth medium was replaced with serum-free medium. Cell cycle distribution was determined by FACS analysis of trypsinized cells after washing with PBS, and resuspension in Triton X-100/ propidium iodide (PI) staining solution as described previously (Krishan, 1975).

### Determination of Intracellular ATP Content

A multiplexed luminescent cell assay was used to measure cellular ATP levels and these measurements were normalized to the number of cells. CellTiter-Fluor^TM^ Cell Assay (Promega) was carried out preceding ATP measurement. The assay is based on the principle that the cell-permeable fluorogenic peptide substrate GF-AFC (glycylphenylalanyl-aminofluorocoumarin) enters only intact cells, and is cleaved by cell-associated protease activity. This generates a fluorescent signal proportional to the number of viable cells. The non-toxic nature of the GF-AFC substrate makes it suitable for multiplexing with the subsequent CellTiter-Gloaaa Luminescent assay to measure ATP levels.

Assays were carried out using a sequential protocol. Cells were seeded in a 96-well plate in triplicates in 100 μl media. First, Cell Titer-Fluor reagent that contains substrate GF-AFC was added to generate fluorescent signal proportional to the constitutive protease activity within viable cells. Fluorescence was measured using a fluorescence reader set up at 380–400 nm excitation, 505 nm emission. Next, CellTiter-Glo^®^ Reagent that contains substrate luciferin was added to the cells. Following cell lysis, a luminescent signal proportional to the amount of ATP present in the cells was generated. Luminescence was measured using a Veritas luminometer. Analyses were performed in biological triplicates and results normalized to the number of cells.

### NF-κBp65 Transcription Factor Assay

The DNA-binding activity of NF-κBp65 protein was measured using the EZ-Detect chemiluminescent TF assay kit (Pierce, 89859, Rockford, IL) following the manufacturer’s instructions. Briefly, this assay measures binding of NF-κB present in nuclear extracts to immobilized NF-κB65 DNA binding-consensus sequences. 10 μg of nuclear extract were prepared according to the manufacturer’s protocol and incubated with 50 μl of binding buffer for an hour with mild agitation at room temperature. NF-κBp65 bound to plates was detected using an NF-κBp65 primary antibody followed by HRP-conjugated secondary antibody incubations for an hour each at room temperature. Chemiluminescence, generated by adding equal amounts of Luminol/Enhancer Solution and Stable Peroxide Solution, was immediately measured using a Veritas Microplate Luminometer with an integration time of 8 s. Luminometric data were expressed as relative light units and converted to fold-change relative to the control samples. Assays were performed in biological triplicates.

### Crystal Violet Viability Assay

Cells were seeded in 96-well microplates at a density of 5000 cells/well. Cells were washed twice with 1XPBS and fixed using 70% ethanol for 10 min at room temperature. The ethanol was then discarded and the plate was dried briefly. The cells were stained with crystal violet solution (0.2% crystal violet in 2% (v/v) ethanol) for 20 min at room temperature. The stained cells were subsequently washed thrice with 1XPBS. Finally, the crystal violet was solubilized in 1% SDS for 1 h on a shaker at room temperature. Absorbance was measured at 595 nm with a spectrophotometric plate reader.

### Bioinformatics Data Analysis

RNA was isolated from the cell lysate using Qiagen RNeasy mini kit according to the manufacturer’s instructions. Gene expression analysis for the experiment was performed with the Illumina platform (Illumina Corp. San Diego, CA, USA), using Human V6 arrays with 47323 transcripts. The raw data was deposited at the NCBI Gene Expression Omnibus (GSE67981). Data sets were normalized and outliers were excluded. Fold changes between four treated samples from two experiments relative to two samples from a control were determined, further filtered by a *t*-test between readouts and removal of unknown/predicted transcripts. From the resulting list of 2310 transcripts (fold changes >2, *p* < 0.005) only 75 of the highest induced and repressed transcripts (fold changes >3.4) were selected. The reduction to 150 transcripts, representing 0.3% of all differentially expressed genes, provides a focus on biologically relevant changes and minimizes false-discovery-rates (Robinson et al., 2010). Promoter regions between -450 and +50 bp of these genes were scanned by Pscan (Zambelli et al., 2009). Enrichment significance scores (*p*-values) reflect likelihoods for specific TFs to bind to promoters with respect to the whole genome promoter set, based on a *z* test (Zambelli et al., 2009). Each TF is characterized by a motif or profile, as provided in the database JASPER, 2014 built (Portales-Casamar et al., 2010). A total of 263 motifs are available in JASPAR. TF motifs scores with low *p*-values are considered enriched, and motifs with high *p*-values are considered avoided. The regulatory TF network was inferred by connectivity data available from DNaseI footprints for fibroblast cell line AG10803, used for our experiments (Neph et al., 2012).

## Results

### Characterization of the ‘Energy Restriction’ Model

Exposure of fibroblasts to culture medium devoid of serum and growth factors for 24 h rendered these cells quiescent. This was confirmed by cell cycle analysis revealing that the fraction of cells in G0/G1 phase increased from 45 to 90% (see Supplementary Table 1).

Quiescent cells exhibited slightly reduced ATP levels as compared to proliferating cells (Supplementary Figure S2). To reduce ATP levels even further, additional metabolic inhibitors were added to the culture medium for a period of 24 h. Pharmacological inhibition of mitochondrial respiration alone reduced intracellular ATP concentrations only moderately when uncouplers (DNP, FCCP) were used at concentrations compatible with long-term (24 h) cell survival. Similar results were obtained when the glucose uptake inhibitor 2-deoxy-glucose (2DG) was applied. However, combining mitochondrial respiration inhibitors and 2DG markedly reduced total ATP content to about 5% of untreated controls (Supplementary Figure S2). At ATP concentrations beyond 80%, cell shrinkage became visually noticeable at 24 h (at 10× magnification), which was reversed within another 24 h and did not cause loss of cell viability or attachment as assessed by crystal violet stain (data not shown). Based on these findings we used a combination of 5 mM DNP and 5 mM 2DG for all subsequent experiments.

### Effects of Nutrient and Energy Deprivation on Transcription Factor Networks in Quiescent Fibroblasts

**FIGURE 1:**
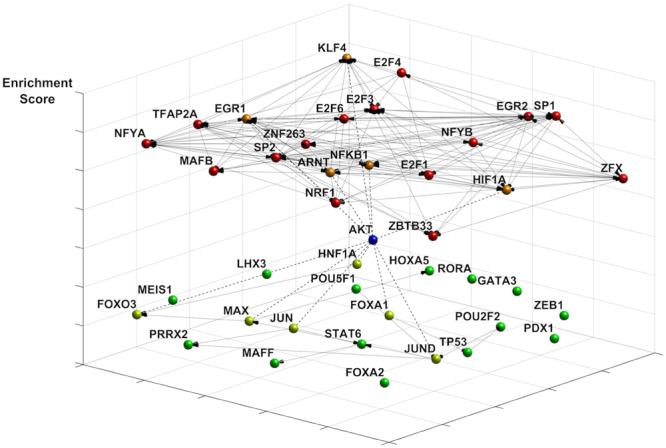
**Effects of ATP restriction on gene regulatory networks in quiescent fibroblasts.** The transcription factor (TF) network was constructed using the most enriched TFs motifs (JASPAR 2014 database). Co-regulatory connections for these motifs are provided by DNaseI footprinting (see text). Represented are the most enriched (*p* < 0.005, coded in red) and avoided (*p* > 0.998, coded in green) motifs, whereby *p*-values indicate the likelihood for a motif to occur against the background of 263 promoters in all known genes. Enriched motifs indicate a highly connected network (20 nodes, 127 edges), with many highly connected nodes including the NF-κB TF binding motif NFKB1. The network of avoided TF motifs is only sparsely connected (20 nodes, 16 links). The differentiation of the motifs by energy restriction predicts a role of Akt signaling in shaping changes in gene expression. Akt connects to TFs in the enriched group (dotted line, orange), such as EGR1, KLF4, and NFKB1. TFs known to be inhibited by Akt include FOXO TFs, HNF1A, and JUN (dotted line, neon green).

We investigated changes in the transcriptional landscape associated with energy restriction imposed by nutrient and ATP restriction. Since TFs can both activate and repress gene expression, based on cellular context and the dynamics of TF interactions (Cusanovich et al., 2014), we focused on the top 150 transcripts that were either up- or downregulated (Supplementary Data Sheet 1). We identified enriched TF motifs using the JASPAR 2014 database whereby *p*-values indicate the likelihood for a motif to occur against the background of promoters of all known genes (Mathelier and Wasserman, 2013). **Figure 1** shows the 20 most enriched and avoided TFs present in those genes out of 263 (based on *p*-value ranking, at *p* < 0.005 and *p* > 0.998 cutoffs, respectively) and their co-regulatory network for which connectivity information was available by DNaseI footprinting (Neph et al., 2012). Included in this group are highly connected nodes such as TFs SP1, SP2, KLF5, and NFKB1, one of the DNA binding motifs of NF-κB protein complexes modeled in JASPAR (JASPAR ID = MA0105.1; Grilli et al., 1993). The sparse co-regulatory network of avoided TFs was not substantially increased in its connectivity when TP53, also an avoided TF (*p* = 0.983), was added. A subset of TF proteins is associated with Akt, as predicted by STRING (Jensen et al., 2009). This includes EGR1, NFKB1 (Cheng and Kane, 2013), and HIF1A (Arsham et al., 2004) in the enriched group as well reduced levels of forkhead TFs (FOXA1, FOXO3) (Brunet et al., 1999; Zhang et al., 2011) and p53 (Mayo and Donner, 2001) in the avoided group. Interestingly, TFs of the E2F group (E2F1, E2F3, E2F4, E2F6) which are known to activate Akt (Chaussepied and Ginsberg, 2004) are also among the top enriched TFs. Collectively, this analysis points to coordinate alterations in NF-κB, Akt and p53 signaling nodes in quiescent fibroblasts undergoing energy restriction.

### Energy Restriction and Activation of Adaptive Signaling Pathways

**FIGURE 2:**
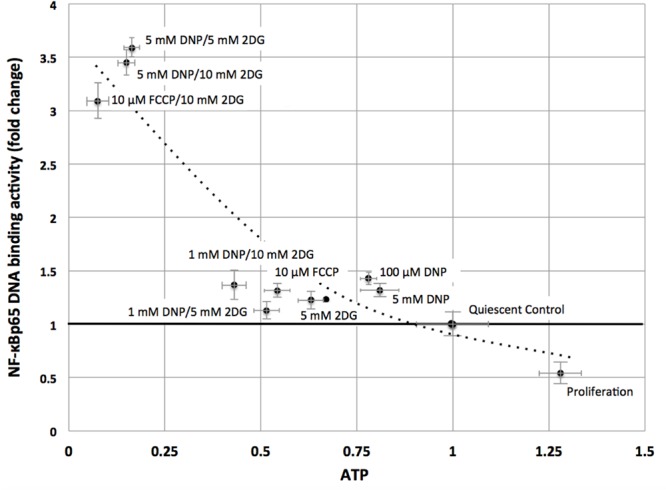
**NF-κBp65 binding activity levels are inversely correlated with intracellular ATP levels in quiescent fibroblasts.** ATP concentrations were reduced following treatment of mitochondrial uncouplers (DNP, FCCP) and/or glycolysis inhibitor (2DG) at different concentrations as indicated. NF-κBp65 DNA binding activity was moderately increased following separate treatment with mitochondrial uncoupler (DNP/FCCP) or glycolysis inhibitor (2DG), or low concentrations of combined inhibitors. Marked increase in NF-κBp65 DNA binding activity occurred for combined treatment with mitochondrial uncouplers (DNP/FCCP) and glycolysis inhibitor (2DG) at higher concentrations. Results shown represent means and standard deviations from three independent experiments, and the best fit between ATP concentrations and NF-κBp65 DNA binding activity is an exponential function (NF-κB = 3.52 e^-1.43ATP^, *R*^2^ = 0.86).

**FIGURE 3:**
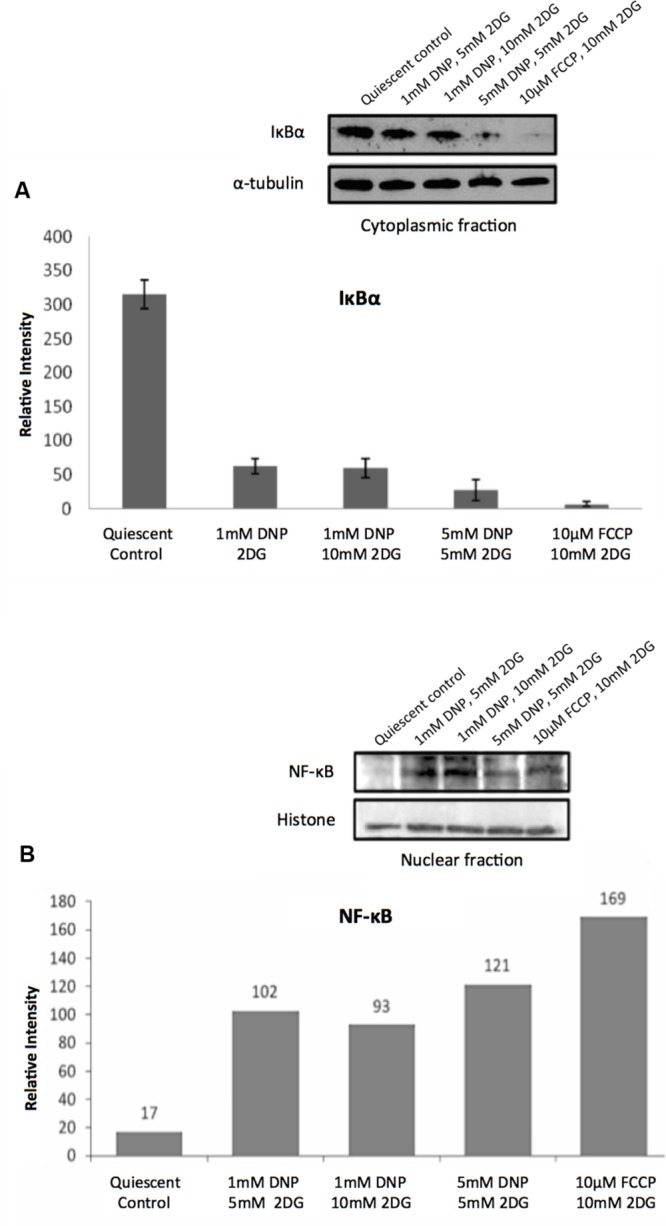
**Regulation of NF-κB activity in response to energy starvation. (A)** Effects of glycolysis inhibitors and mitochondrial uncouplers on IκBα expression levels in cytoplasmic fractions. The bar graph shows results of densitometric analysis of Western blot band intensities normalized to α-tubulin expression (means and standard deviations from three independent experiments). The differences between the control and treatments are significant (*t*-test, *p* < 0.001). **(B)** Effects of glycolysis inhibitors and mitochondrial uncouplers on NF-κBp65 (phospho S276) levels in the nuclear fractions. Densitometric analysis of Western blot band intensities shown are normalized to histone loading control.

Consistent with the TF analysis progressive energy restriction was accompanied by a substantial increase in NF-κB activity as determined by NF-κBp65 DNA binding, which was highest in cells subjected to combined 5 mM DNP and 5 mM 2DG treatment (**Figure 2**) and returned to baseline levels within 24 h after removing metabolic inhibitors (Supplementary Figure S3). Increased NF-κB activity was further associated with reduced IκBα levels in cytoplasmic extracts of energy-challenged cells, suggesting activation of the canonical NF-κB signaling pathway under these conditions (**Figure 3A**). Furthermore, concomitant treatment of fibroblasts with inhibitors of mitochondrial respiration and glycolysis increased the abundance of transcription-competent NF-κBp65 phosphorylated on serine 276 (**Figure 3B**). In aggregate, these observations suggest that, in quiescent fibroblasts, ATP levels and NF-κB activity are inversely correlated.

**FIGURE 4:**
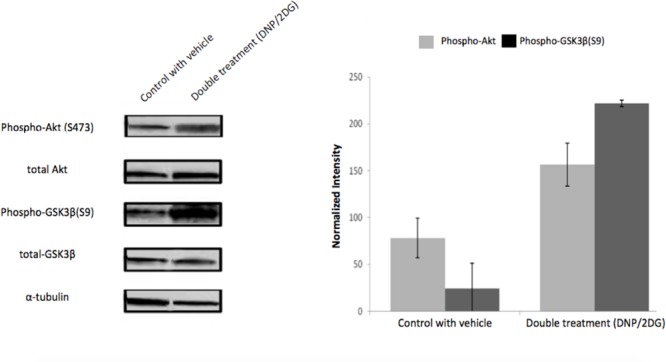
**Effects of metabolic inhibitors on Akt phosphorylation.** Increased phosphorylation of Akt and its substrate GSK3-β was observed upon DNP/2DG treatment at concentrations of 5 mM each. A representative blot is shown. The bar graphs represent means and standard deviations of three experiments. The difference between control and treated samples in Akt (S473) phosphorylation is weakly significant (*t*-test, *p* < 0.1) and phosphorylation of GSK3β (S9) significant (*t*-test, *p* < 0.005). Images for each protein originate from the same gel, cropped to show only two lanes (control and DNP/2DG treatment).

**FIGURE 5:**
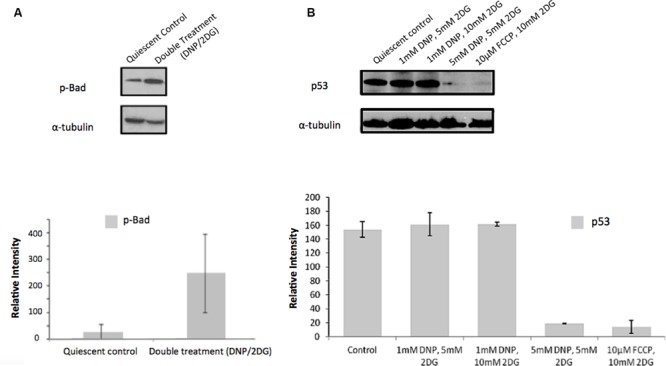
**Effect of metabolic inhibitors on Bad and p53. (A)** Increased Bad phosphorylation upon DNP/2DG treatment at concentrations of 5 mM each. The change is not significant. **(B)** Decreased p53 expression upon treatment with different inhibitors and concentrations. Results of densitometric analysis of immunoblots shown are normalized to the α-tubulin loading control. Means and standard deviations of three experiments are shown. The change is significant if compared to the control at concentrations of 5 nM DNP/5 mM 2DG and 10 μm FCCP/ 10 mM 2DG, (*t*-test, *p* < 0.001).

Similarly, and further consistent with the TF analysis, ATP deprivation had distinct effects on the functional state of Akt. Specifically, reduction of intracellular ATP levels was associated with increased Akt phosphorylation at Ser-473 and of the Akt target glycogen synthase kinase (GSK3β) at Ser-9 (**Figure 4**). GSK3β is a multifunctional serine-threonine kinase and inhibition of its activity by way of Ser-9 phosphorylation can enhance cell survival and NF-κB activation (Hoeflich et al., 2000) and reduce apoptosis (Pap and Cooper, 1998). In addition, energy restriction was associated with increased Bad phosphorylation at Ser-132 (**Figure 5A**). Consistent with the changes in TF abundance we observed that energy restriction was associated with decreased p53 protein abundance (**Figure 5B**). In combination, these effects may contribute to reduced apoptosis susceptibility, dissociation of Bad/p53 complexes and association of Bad with Bcl-2 and Bcl-xL (Datta et al., 1997; Jiang et al., 2006).

**FIGURE 6:**
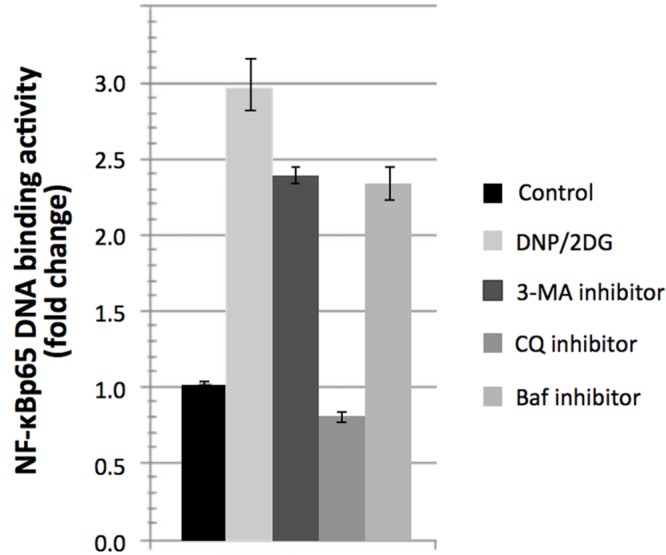
**Reversal of NF-κBp65 DNA binding activity levels associated with DNP/2DG treatment by autophagy inhibitors.** Reversal in NF-κBp65 DNA binding activity following treatment with autophagy inhibitors 3-methyladenine (3-MA), chloroquine (CQ), and Bafilomycin-A1 (Baf). Means and standard deviations of three independent experiments are provided. For all inhibitors, NF-κBp65 DNA binding activity is significantly reduced if compared to the DNP/2DG treated sample (*t*-test, *p* < 0.005).

Interestingly, treatment with DNP and 2DG inhibitors downmodulated mTOR activity as indicated by increased phosphorylation of eIF-2alpha at serine 52 and decreased p70-S6K phosphorylation at threonine 389 (Supplementary Figure S4). This is at variance with mTOR activation downstream of Akt yet consistent with the view that ATP restriction leads to a compensatory switch to autophagy. Autophagy induction was indicated by increased conversion of LC3B-I–LC3B-II, a marker of autophagy under energy stress, increased ATG5 expression levels and reduced levels of p62 (Supplementary Figure S5). We sought to investigate if induction of autophagy itself influences NF-κB activation during energy depletion (using a combination of 5m M DNP and 5m M 2DG). Autophagy inhibition was accomplished by using three different pharmacological inhibitors, 3-Methyladenine (3-MA), Chloroquine (CQ), and Bafilomycin-A1 (Baf). 3-MA is known to inhibit class III PI3K involved in the autophagosome formation and the other two inhibitors halt the fusion of autophagosomes with lysosomes. Autophagy inhibition in energy-restricted cells resulted in decreased NF-κBp65 DNA binding activity, which was significant for CQ, as shown in **Figure 6**, which suggested that autophagy induction is one of the cellular processes contributing to NF-κB activation in cells encountering reduced ATP levels. Collectively, the integration of signaling nodes with roles in cell fate decisions within the regulatory network points to a complex and finely tuned stress response.

## Discussion

Here we report that metabolic stress caused by nutrient deprivation and restricted ATP production leads to marked changes in signaling networks in quiescent fibroblasts. Consistent with previous reports, withdrawal of exogenous growth factors induced a quiescent state with only marginal effects on ATP production (Boraldi et al., 2008; Lemons et al., 2010). To investigate the compensatory response of resting cells to ATP restriction we assessed the consequences of dual inhibition of glucose transport and mitochondrial uncoupling on TF networks of quiescent fibroblast. Similar experimental approaches to ATP restriction have been previously been taken to study the effect of ischemia in myocytes and neurons (Rose et al., 1998; Murphy and Steenbergen, 2008). As reported here, restricting ATP production induced broad changes in the TF network consistent with several connected signaling nodes.

Perhaps the most salient finding of this study is the observation that ATP deprivation in quiescent cells is associated with reversible activation of the canonical NF-κB pathway. This finding dovetails nicely with an emerging theme pointing to metabolic control of signaling pathways with established roles in the context of inflammation and immunity (Tornatore et al., 2012). In addition to the TF NF-κB signature we observed that energy deprivation by mitochondrial uncoupling increased NF-κBp65 binding activity levels 1.5-fold. The addition of glucose uptake inhibitors, reducing ATP levels to 1/10th of the level observed in quiescent cells, further increased NF-κBp65 binding activity levels to approximately 3-fold.

It is poorly understood which signals and pathways contribute to enhanced NF-κB activity in ATP-deprived cells. It is possible that autophagy and, potentially, lysosomal degradation of IκB (Cuervo et al., 1998; Qing et al., 2006) participate in the process. However, since NF-κB pathway activation is likely a composite response integrating multiple stressors and danger signals, it is likely that additional mechanisms contribute to its initiation or potentiation in response to the metabolic challenges (Boraldi et al., 2008; Caldwell et al., 2014; Fujita et al., 2016). NF-κB activation is commonly considered to represent an adaptive mechanism in support of cell survival and resistance to various cell stresses (Van Antwerp et al., 1998; Karin and Lin, 2002). Reduced ATP abundance and attendant upregulation of NF-κB signaling has been reported in aging and in a wide spectrum of diseases. For example, energy starvation is a hallmark of cardiac injury (Ingwall and Weiss, 2004) and NF-κB signaling has been implicated in myocyte survival (Gordon et al., 2011). Similarly, the activation of stress signaling pathways including NF-κB has been associated with alterations in mitochondrial function and glucose transport in diabetes and neuronal pathologies (Wellen and Hotamisligil, 2005; Shih et al., 2015).

In addition to NF-κB activation, we describe activation of Akt-dependent signaling in support of cell survival as evidenced by the TF signature analysis as well as phosphorylation of Akt and its targets Bad and GSK3β. Inactivation of GSK3β reportedly increases the apoptotic threshold specifically for intrinsic mitochondrial stress (Beurel and Jope, 2006). It remains to be investigated whether Akt-dependent signaling events contribute to NF-κB activation in energy-challenged cells. Consistent with this view, Akt has previously been shown to activate NF-κB and other TFs in other experimental settings (Bai et al., 2009). Interestingly, Akt activation is ‘selective’ in the sense that it encompasses several elements of the Akt transcript signature but does not extend to increased mTOR signaling. In contrast, mTOR activity is reduced in nutrient and energy-deprived cells most likely as part of the catabolic and autophagic state of glucose-starved, ATP-deprived cells.

Furthermore, p53 was expressed at reduced transcript and protein levels in ATP-deprived cells. This finding contrasts with enhanced p53 expression commonly observed in cells exposed to genotoxic or proteotoxic stress and in senescent cells (Itahana et al., 2001; Riley et al., 2008). Downmodulation of p53 expression levels may be, in part, contributed to by Akt as Akt enhances Mdm2-mediated ubiquitination and degradation of p53 (Ogawara et al., 2002). The significance of this observation as it relates to the setting of apoptotic thresholds in quiescent cells, and to the accumulation of DNA damage in long-term quiescent cell cultures (Marthandan et al., 2014), remains to be determined.

## Conclusion

The experimental approach taken in this study introduces a facile model to reveal targets, mechanisms and molecular network adjustments reflective of adaptive cell stress responses and extending beyond previously known pathways. While previous work largely focused on signals associated with cell death mechanisms triggered by energy starvation (Simm et al., 1997; Lieberthal et al., 1998; Leicht et al., 2001; Graham et al., 2012) we identified a TF signature which, in aggregate, is likely to contribute to cell survival under challenging metabolic conditions associated with marked restriction of ATP production. This signature involves key signaling nodes associated with inflammation and genome maintenance beyond canonical pathways previously linked to energy sensing and stress responses such as Insulin/IGF, sirtuins, mTOR, and AMPK signaling (Haigis and Yankner, 2010; Kenyon, 2010). The relative importance of individual signaling components as they relate to successful adaption of quiescent cells to energy challenge remains to be investigated.

## Author Contributions

AK, NY, CK, and UR planned experiments, NY and DA performed experiments, NY, KD, DA, and AK performed data analysis, AK, NY, and UR wrote the manuscript, AK, CK, and UR supervised the study.

## Conflict of Interest Statement

The authors declare that the research was conducted in the absence of any commercial or financial relationships that could be construed as a potential conflict of interest.
