# Supplementary material for: Distinct Cell Stress Responses Induced by ATP Restriction in Quiescent Human Fibroblasts
Source: Front Genet. 2016 Oct 4;7:171. doi: 10.3389/fgene.2016.00171 (PMC5047886; doi:10.3389/fgene.2016.00171)
Supplement: Supplementary file 1 [file Table_1.PDF]

**Supplemental Table 1**

| Phase | + FCS | -FCS (24h) |
|---|---|---|
| %G0/G1 | 45.3 | 95.0 |
| %G2/M | 31.3 | 3.07 |
| % S | 21.7 | 0.83 |
| % APOP | 0.96 | 0.95 |
| % APOP and Necro | 2.06 | 1.12 |

Cell cycle analysis of fibroblast cell line AG 10803, before and after 24h withdrawal of fetal calf serum (FCS). Percentages of cells in different cell cycle phases was determined by FACS (BD FACS Calibur) analysis of propidium iodide-stained cells.
