# Supplementary material for: Distinct Cell Stress Responses Induced by ATP Restriction in Quiescent Human Fibroblasts
Source: Front Genet. 2016 Oct 4;7:171. doi: 10.3389/fgene.2016.00171 (PMC5047886; doi:10.3389/fgene.2016.00171)
Supplement: Supplementary file 2 [file Data_Sheet_1.pdf]

Supplemental Data Sheet I

Genes	Ranking of Transcription Factor Enrichment Scores		
**Accession#**	**Motif**	**Name**	**p-value**
NM_005542	MA0516.1	SP2	4.10E-08
NM_000527	MA0599.1	KLF5	1.36E-07
NM_000090	MA0079.3	SP1	2.15E-07
NM_003246	MA0162.2	EGR1	5.48E-07
NM_005891	MA0079.2	SP1	9.04E-07
NM_002048	MA0060.1	NFYA	1.58E-06
NM_005602	MA0039.2	Klf4	2.84E-06
NM_133468	MA0502.1	NFYB	1.37E-05
NM_006206	MA0470.1	E2F4	1.59E-05
NM_002130	MA0060.2	NFYA	3.14E-05
NM_005063	MA0024.2	E2F1	5.45E-05
NM_015419	MA0006.1	Arnt::Ahr	0.000114019
NM_013282	MA0162.1	Egr1	0.000209794
NM_153000	MA0117.1	Mafb	0.00026863
NM_015441	MA0506.1	NRF1	0.000295573
NM_019035	MA0471.1	E2F6	0.000327775
NM_014762	MA0003.1	TFAP2A	0.000425223
NM_145693	MA0472.1	EGR2	0.000518135
NM_001548	MA0469.1	E2F3	0.000547165
NM_006206	MA0493.1	Klf1	0.000651229
NM_032291	MA0259.1	HIF1A::ARNT	0.000745246
NM_145260	MA0105.2	NFKB1	0.00109283
NM_001850	MA0163.1	PLAG1	0.00145191
NM_022469	MA0527.1	ZBTB33	0.00206305
NM_003485	MA0056.1	MZF1_1-4	0.00217672
NM_019035	MA0048.1	NHLH1	0.00256759
NM_080927	MA0528.1	ZNF263	0.00462619
NM_002546	MA0079.1	SP1	0.00463975
NM_012242	MA0146.2	Zfx	0.00488199
NM_032623	MA0146.1	Zfx	0.00494622
NM_177403	MA0105.1	NFKB1	0.00671815
NM_199168	MA0057.1	MZF1_5-13	0.00792507
NM_004104	MA0524.1	TFAP2C	0.00881441
NM_080927	MA0513.1	SMAD2::SMAD3::SMAD4	0.01012
NM_018689	MA0003.2	TFAP2A	0.0108328
NM_003713	MA0119.1	TLX1::NFIC	0.0131541
NM_000859	MA0149.1	EWSR1-FLI1	0.0164912

NM_003247	MA0131.1	HINFP	0.0250658
NM_016095	MA0116.1	Zfp423	0.0307509
NM_002185	MA0143.3	Sox2	0.0341472
NM_002340	MA0597.1	THAP1	0.0350127
NM_006009	MA0018.1	CREB1	0.036123
NM_032623	MA0500.1	Myog	0.0389382
NM_004508	MA0073.1	RREB1	0.0391393
NM_206929	MA0024.1	E2F1	0.040873
NM_001124	MA0155.1	INSM1	0.04575
NM_207645	MA0515.1	Sox6	0.0491967
NM_177414	MA0139.1	CTCF	0.0629898
NM_021111	MA0161.1	NFIC	0.0668415
NM_198336	MA0147.1	Myc	0.0692172
NM_152314	MA0107.1	RELA	0.0703128
NM_206930	MA0100.1	Myb	0.0772717
NM_003500	MA0595.1	SREBF1	0.0804987
NM_016613	MA0028.1	ELK1	0.088127
NM_031243	MA0104.2	Mycn	0.0918047
NM_002780	MA0035.1	Gata1	0.104258
NM_030967	MA0521.1	Tcf12	0.105588
NM_012093	MA0105.3	NFKB1	0.1061
NM_020351	MA0514.1	Sox3	0.109191
NM_032623	MA0076.1	ELK4	0.10997
NM_173485	MA0154.2	EBF1	0.11213
NM_020954	MA0504.1	NR2C2	0.133942
NM_002048	MA0014.1	Pax5	0.134519
NM_177964	MA0112.1	ESR1	0.164042
NM_004462	MA0600.1	RFX2	0.172287
NM_014573	MA0101.1	REL	0.2053
NM_012098	MA0154.1	EBF1	0.205699
NM_006195	MA0112.2	ESR1	0.227007
NM_030966	MA0159.1	RXR::RAR_DR5	0.23257
NM_000903	MA0596.1	SREBF2	0.255062
NM_018153	MA0591.1	Bach1::Mafk	0.259728
NM_001098816	MA0499.1	Myod1	0.262601
NM_053064	MA0510.1	RFX5	0.275413
NM_014220	MA0133.1	BRCA1	0.307374
NM_139274	MA0509.1	Rfx1	0.31067
NM_021111	MA0095.1	YY1	0.325347
NM_001076552	MA0522.1	Tcf3	0.325688
NM_001146	MA0062.2	GABPA	0.326619
NM_001017425	MA0077.1	SOX9	0.338824

NM_002514	MA0017.1	NR2F1	0.372696
NM_016498	MA0145.1	Tcfcp2l1	0.374905
NM_178815	MA0145.2	Tcfcp2l1	0.379936
NM_015599	MA0039.1	Klf4	0.386517
NM_004823	MA0062.1	GABPA	0.407565
NM_021958	MA0059.1	MYC::MAX	0.41992
NM_003486	MA0014.2	PAX5	0.421515
NM_152270	MA0138.1	REST	0.443049
NM_004793	MA0164.1	Nr2e3	0.4482
NM_002056	MA0108.2	TBP	0.459337
NM_004675	MA0076.2	ELK4	0.466329
NM_022117	MA0598.1	EHF	0.4718
NM_203472	MA0114.1	HNF4A	0.477416
NM_000407	MA0108.1	TBP	0.48707
NM_000600	MA0477.1	FOSL1	0.507674
NM_018420	MA0080.1	SPI1	0.508298
NM_005851	MA0078.1	Sox17	0.520085
NM_201525	MA0065.2	PPARG::RXRA	0.556062
NM_014637	MA0518.1	Stat4	0.559765
NM_014445	MA0069.1	Pax6	0.573767
NM_001012661	MA0058.1	MAX	0.57435
NM_001025366	MA0004.1	Arnt	0.586671
NM_019089	MA0525.1	TP63	0.598056
NM_001012974	MA0111.1	Spz1	0.604282
NM_033452	MA0137.1	STAT1	0.61433
NM_021147	MA0484.1	HNF4G	0.62853
NM_152331	MA0007.1	Ar	0.633919
NM_015641	MA0150.2	Nfe2l2	0.645647
NM_004794	MA0104.3	Mycn	0.654059
NM_006636	MA0592.1	ESRRA	0.65769
NM_019096	MA0098.1	ETS1	0.661672
NM_022117	MA0083.2	SRF	0.664631
NM_001040409	MA0066.1	PPARG	0.689169
NM_152829	MA0144.2	STAT3	0.697615
NM_016498	MA0478.1	FOSL2	0.711773
NM_002056	MA0114.2	HNF4A	0.726637
NM_002201	MA0144.1	Stat3	0.727306
NM_184237	MA0494.1	Nr1h3::Rxra	0.733484
NM_032431	MA0517.1	STAT2::STAT1	0.736554
NM_018177	MA0490.1	JUNB	0.737267
NM_182491	MA0067.1	Pax2	0.744455
NM_020127	MA0482.1	Gata4	0.74862

NM_004183	MA0065.1	PPARG::RXRA	0.751701
NM_013388	MA0442.1	SOX10	0.758452
NM_017786	MA0258.1	ESR2	0.761602
NM_001717	MA0486.1	HSF1	0.763083
NM_015525	MA0088.1	znf143	0.774489
NM_152995	MA0093.1	USF1	0.780295
NM_001013251	MA0137.3	STAT1	0.790985
NM_173583	MA0143.2	Sox2	0.800372
NM_003324	MA0143.1	Sox2	0.801136
NM_004864	MA0467.1	Crx	0.801647
NM_004675	MA0103.2	ZEB1	0.808427
NM_004733	MA0074.1	RXRA::VDR	0.809681
NM_004793	MA0099.2	JUN::FOS	0.820912
NM_003376	MA0083.1	SRF	0.821912
NM_006134	MA0141.1	Esrrb	0.826545
NM_139314	MA0258.2	ESR2	0.827443
NM_017947	MA0508.1	PRDM1	0.828853
NM_032239	MA0130.1	ZNF354C	0.834276
NM_012162	MA0081.1	SPIB	0.837009
NM_005384	MA0141.2	Esrrb	0.837304
NM_015359	MA0481.1	FOXP1	0.84834
NM_024111	MA0503.1	Nkx2-5	0.849362
NM_002220	MA0070.1	PBX1	0.85322
NM_032683	MA0122.1	Nkx3-2	0.856079
NM_021101	MA0106.1	TP53	0.862817
NM_153742	MA0512.1	Rxra	0.865358
NM_024555	MA0475.1	FLI1	0.868614
NM_000600	MA0109.1	Hltf	0.872647
NM_021158	MA0491.1	JUND	0.874004
NM_024866	MA0018.2	CREB1	0.882914
NM_001010990	MA0050.1	IRF1	0.884375
NM_007076	MA0037.2	GATA3	0.889356
NM_005098	MA0473.1	ELF1	0.891325
NM_031479	MA0104.1	Mycn	0.891782
NM_004083	MA0501.1	NFE2::MAF	0.894122
NM_004933	MA0461.1	Atoh1	0.894627
NM_012328	MA0052.2	MEF2A	0.89556
	MA0038.1	Gfi1	0.89879
	MA0050.2	IRF1	0.901176
	MA0102.2	CEBPA	0.902259
	MA0102.1	Cebpa	0.902269
	MA0476.1	FOS	0.907249

MA0019.1	Ddit3::Cebpa	0.908651
MA0138.2	REST	0.908948
MA0137.2	STAT1	0.909864
MA0036.1	GATA2	0.910414
MA0098.2	Ets1	0.914622
MA0035.2	Gata1	0.918683
MA0002.1	RUNX1	0.925927
MA0090.1	TEAD1	0.926909
MA0052.1	MEF2A	0.928244
MA0036.2	GATA2	0.929069
MA0087.1	Sox5	0.929455
MA0489.1	JUN	0.932047
MA0160.1	NR4A2	0.936592
MA0099.1	JUN::FOS	0.936742
MA0497.1	MEF2C	0.938763
MA0089.1	NFE2L1::MafG	0.940617
MA0068.1	Pax4	0.941708
MA0035.3	Gata1	0.945022
MA0051.1	IRF2	0.945454
MA0474.1	Erg	0.946354
MA0007.2	AR	0.949183
MA0115.1	NR1H2::RXRA	0.952564
MA0465.1	CDX2	0.953044
MA0466.1	CEBPB	0.953248
MA0025.1	NFIL3	0.953788
MA0041.1	Foxd3	0.95506
MA0080.2	SPI1	0.956935
MA0080.3	Spi1	0.957019
MA0043.1	HLF	0.957347
MA0462.1	BATF::JUN	0.95806
MA0505.1	Nr5a2	0.958075
MA0152.1	NFATC2	0.964445
MA0480.1	Foxo1	0.964765
MA0136.1	ELF5	0.965265
MA0093.2	USF1	0.965314
MA0002.2	RUNX1	0.968826
MA0150.1	NFE2L2	0.969688
MA0100.2	Myb	0.969995
MA0156.1	FEV	0.973328
MA0147.2	Myc	0.974968
MA0063.1	Nkx2-5	0.975809
MA0102.3	CEBPA	0.979199

MA0084.1	SRY	0.979919
MA0523.1	TCF7L2	0.980633
MA0029.1	Mecom	0.980798
MA0106.2	TP53	0.983238
MA0479.1	FOXH1	0.984638
MA0113.1	NR3C1	0.985502
MA0151.1	ARID3A	0.986182
MA0483.1	Gfi1b	0.988899
MA0593.1	FOXP2	0.989119
MA0140.1	Tal1::Gata1	0.989305
MA0594.1	Hoxa9	0.990444
MA0463.1	Bcl6	0.991642
MA0091.1	TAL1::TCF3	0.991713
MA0485.1	Hoxc9	0.99176
MA0027.1	En1	0.992277
MA0526.1	USF2	0.993391
MA0113.2	NR3C1	0.994058
MA0496.1	MAFK	0.994062
MA0009.1	T	0.99459
MA0095.2	YY1	0.994597
MA0072.1	RORA_2	0.994961
MA0042.1	FOXI1	0.995741
MA0032.1	FOXC1	0.99579
MA0519.1	Stat5a::Stat5b	0.995919
MA0040.1	Foxq1	0.996756
MA0092.1	Hand1::Tcfe2a	0.996801
MA0031.1	FOXD1	0.996868
MA0511.1	RUNX2	0.99698
MA0124.1	NKX3-1	0.997301
MA0148.1	FOXA1	0.997633
MA0148.2	FOXA1	0.997704
MA0033.1	FOXL1	0.997984
MA0464.1	Bhlhe40	0.998082
MA0140.2	TAL1::GATA1	0.998117
MA0071.1	RORA_1	0.998196
MA0125.1	Nobox	0.998375
MA0037.1	GATA3	0.998404
MA0157.1	FOXO3	0.998434
MA0153.1	HNF1B	0.998487
MA0495.1	MAFF	0.998625
MA0046.1	HNF1A	0.998721
MA0520.1	Stat6	0.998942

MA0103.1	ZEB1	0.999123
MA0148.3	FOXA1	0.999185
MA0498.1	Meis1	0.99937
MA0047.2	Foxa2	0.999375
MA0507.1	POU2F2	0.999519
MA0058.2	MAX	0.999541
MA0135.1	Lhx3	0.999559
MA0030.1	FOXF2	0.999572
MA0047.1	Foxa2	0.99964
MA0158.1	HOXA5	0.999663
MA0468.1	DUX4	0.999821
MA0492.1	JUND	0.999876
MA0142.1	Pou5f1::Sox2	0.999932
MA0488.1	JUN	0.999932
MA0132.1	Pdx1	0.999964
MA0075.1	Prrx2	0.999992
