# Supplementary material for: Distinct Cell Stress Responses Induced by ATP Restriction in Quiescent Human Fibroblasts
Source: Front Genet. 2016 Oct 4;7:171. doi: 10.3389/fgene.2016.00171 (PMC5047886; doi:10.3389/fgene.2016.00171)
Supplement: Supplementary file 3 [file Image_1.pdf]

Supplementary Figures

Distinct cell stress responses induced by ATP restriction in quiescent human fibroblasts

Nirupama Yalamanchili, Andres Kriete [*], David Alfego , Kelli M. Danowski, Csaba Kari, Ulrich Rodeck

*Corresponding Author: ak3652@drexel.edu

Supplementary Figure S1

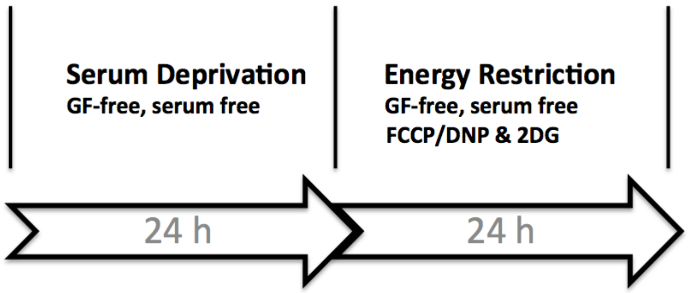

Figure S1: Schematic summary of experimental protocol.

Supplementary Figure S2

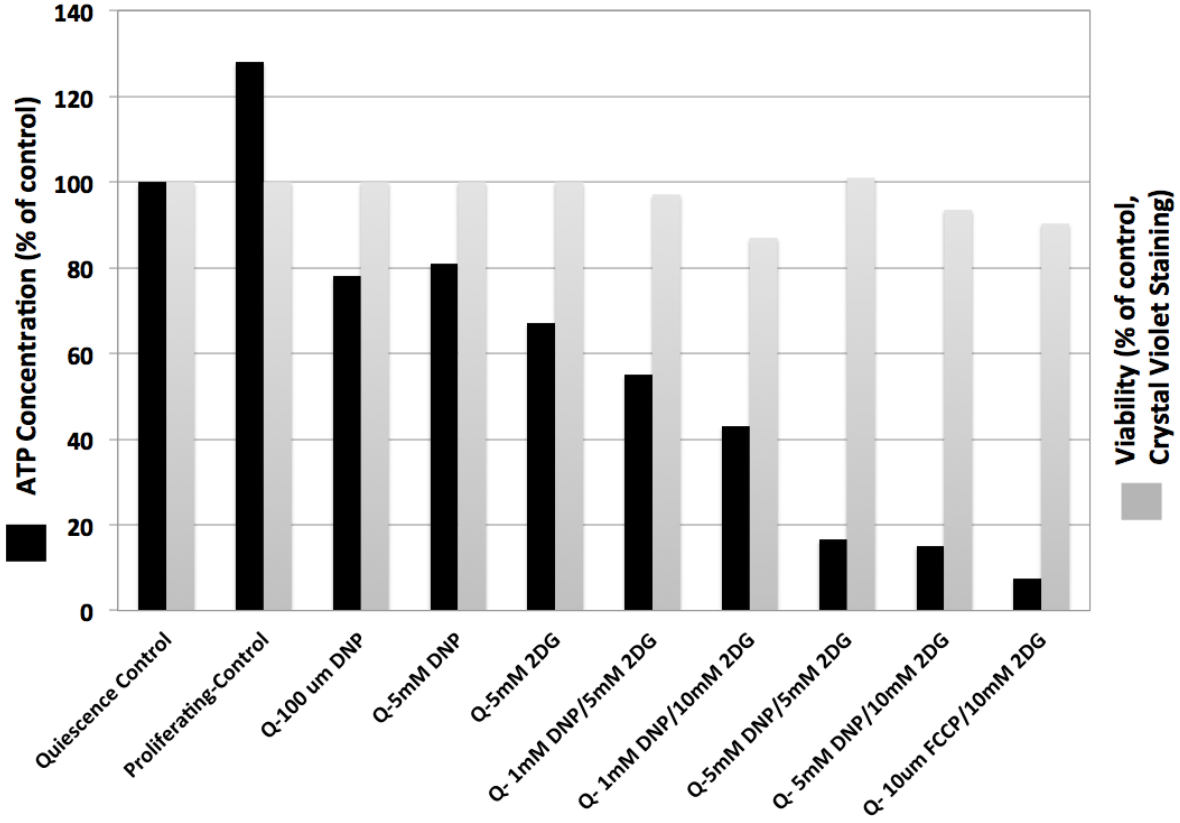

Figure S2. Effects of metabolic inhibitors on ATP concentrations and cell viability. Different concentrations and combinations of the glucose transport inhibitor 2DG and the mitochondrial uncouplers DNP and FCCP were tested. Marked reductions of ATP levels were observed following combined treatments, without compromising cell viability as determined by crystal violet staining.

Supplementary Figure S3

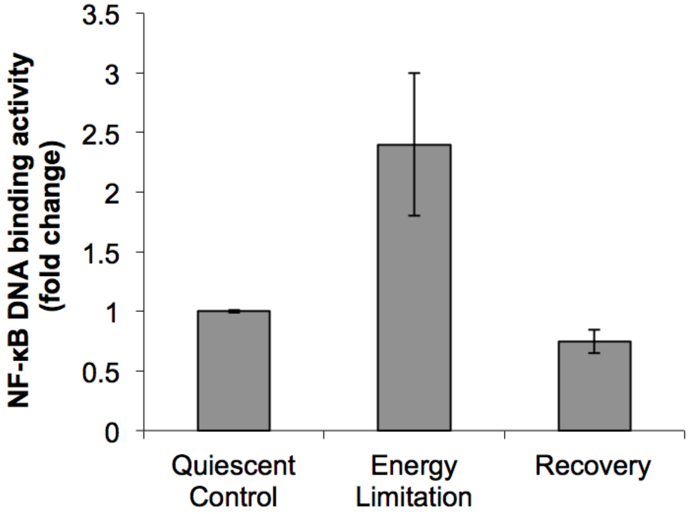

Figure S3: Reversibility of NF-κBp65 DNA binding activity induced by ATP restriction. NF-κBp65 DNA binding activity was determined in cells grown in serum-free, growth factor-free media and treated with DNP/2DG- for 24 h. Recovery of effects on NF-kB activity was determined 4 h after removing DNP/2DG. DNP/2DG-treated cells after 24h energy deprivation, and 4h after removal of DNP/2DG. Results represent means and standard deviations of two independent experiments (t-test , $p<0.05$).

Supplementary Figure S4

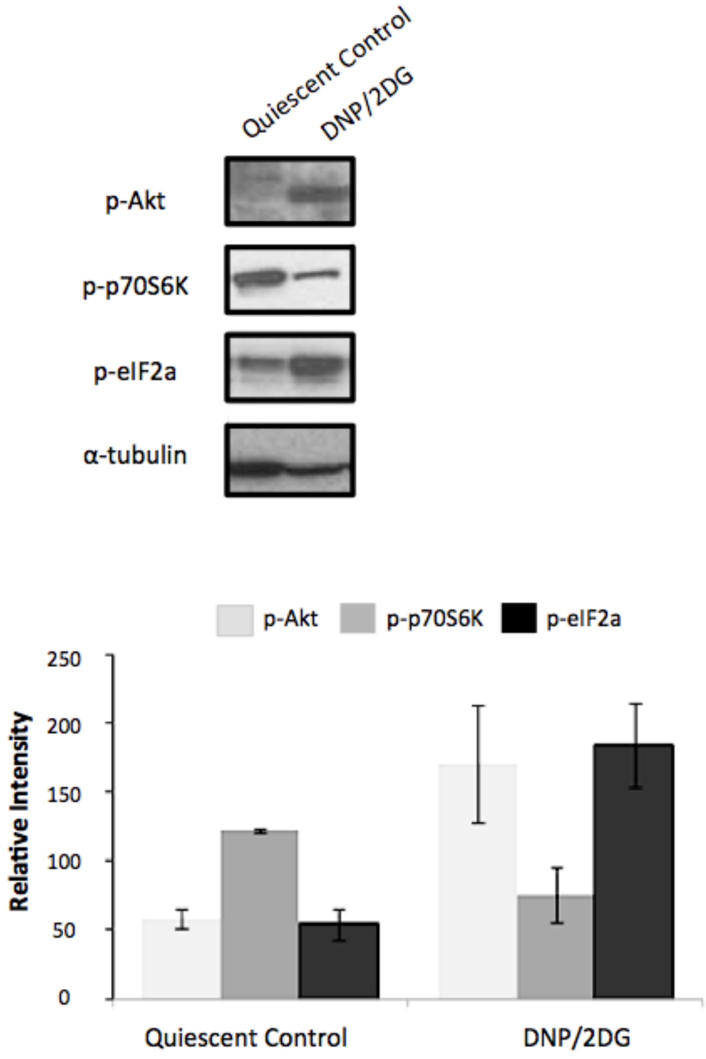

Figure S4: Effects of DNP/2DG (5mM each) on the mTOR targets phospho-p70S6 kinase (Thr389) and phospho-eIF2α (S52). Densitometric analysis of immunoblot band intensities was performed upon normalization to loading control α-tubulin and results shown represent means and standard deviations of three independent experiments. Change in p-Akt and p-p70S6K is weakly significant (t-test, $p<0.1$) and the change of p-eIF2 is significant (t-test, $p<0.05$).

Supplementary Figure S5

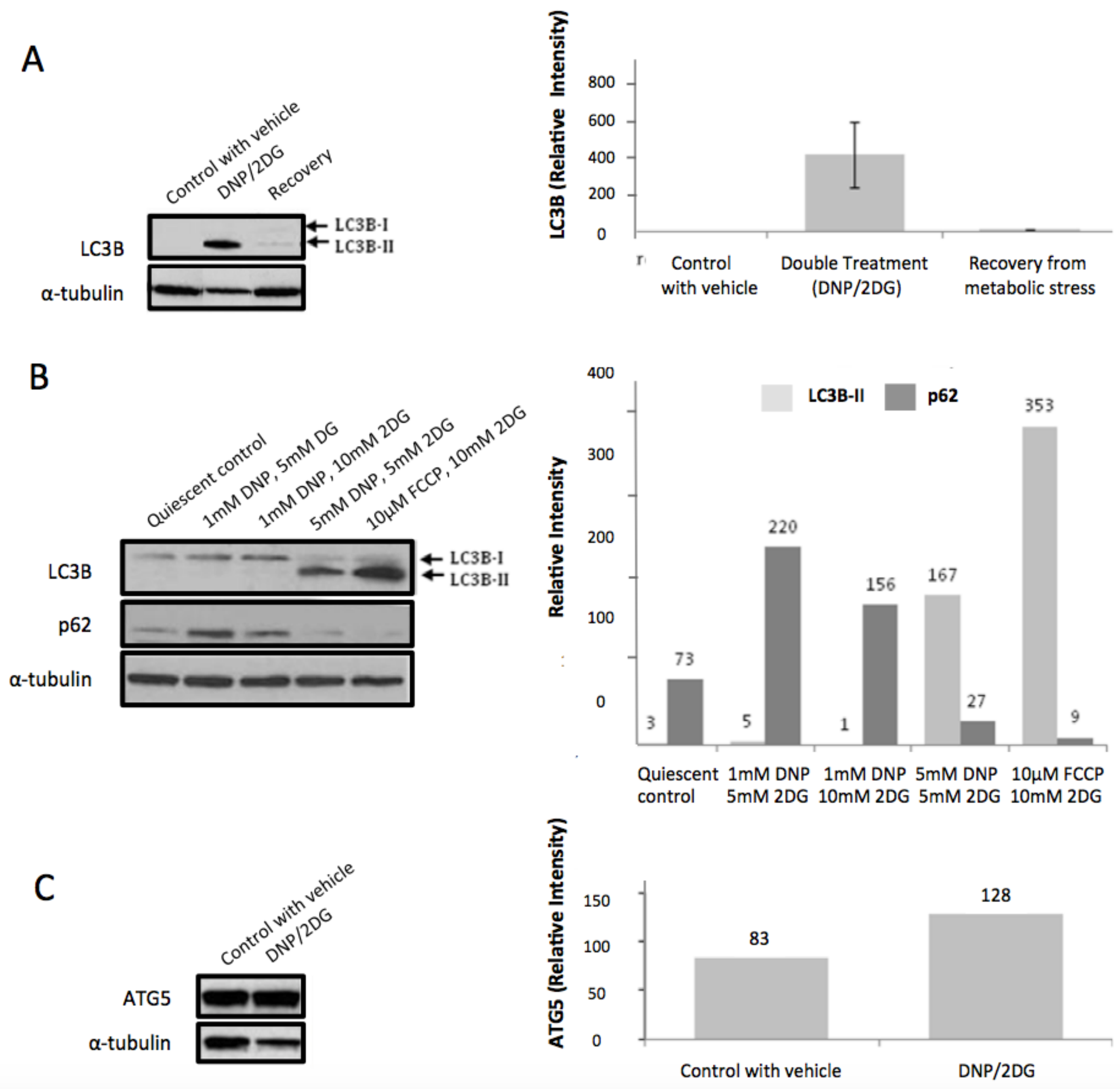

Figure S5: Autophagy induced by ATP restriction. (A) Conversion of the autophagy marker LC3B I to LC3B II upon ATP restriction, relative to quiescent control, which reverts back to normal levels after removal of DNP/2DG. Selected immunoblot bands (from the same gel) are shown on left, and mean intensities are shown on the right, normalized to loading control α-tubulin, with standard deviations from three experiments (t-test, $p<0.05$). (B) Increased conversion of autophagy marker LC3B I to LC3B II and degradation of p62 in the cytoplasmic fractions at increasing doses of DNP/2DG. Immunoblot band intensities normalized to loading control α-tubulin are provided. (C) Effects of DNP/2DG (5mM each) on ATG5 protein expression.
